# BURDEN OF POTENTIALLY INAPPROPRIATE MEDICATIONS AMONG OLDER VETERANS WITH PAIN AND DEMENTIA

**DOI:** 10.1093/geroni/igz038.2632

**Published:** 2019-11-08

**Authors:** Heather Allore, Danijela Gnjidic, Ling Han

**Affiliations:** 1 Yale University, New Haven, Connecticut, United States; 2 The University of Sydney, Sydney, NSW, Australia; 3 Yale School of Medicine, New Haven, Connecticut, United States

## Abstract

Among 115,869 Veterans ≥65 years old with a history of moderate to severe musculoskeletal pain (numerical pain intensity rating ≥4) admitted to Veterans Health Administration hospitals in fiscal year 2013, we tested whether medication use is potentially inappropriate (defined using the 2015 Beers Criteria; categorized as central nervous system (CNS) or other drugs) according to Alzheimer’s Disease and Related Dementias (ADRD) status based on ICD-9 codes. We used Poisson regression to estimate the association of the number of CNS Beers Criteria drugs (anticholinergics, antidepressants, antipsychotics, benzodiazepines, sedative hypnotics, pain medications, and opioids) and other Beers Criteria drugs according to ADRD status. The mean age of the cohort at the index hospital admission was 74.5 (SD 8.2), with 19.0% having ADRD and a Charlson comorbidity index of 3.9±3.0. After adjusting for age, sex, race/ethnicity, marital status, body mass index, 16 diagnostic criteria for pain and chronic conditions, mental health conditions and use of other system Beers Criteria drugs, we found that the mean number of CNS Beers Criteria drugs were 25% higher among Veterans with ADRD, with an adjusted risk ratio (aRR) of 1.25 (95% CI 1.23, 1.27; P <.0001). On the other hand, the mean number of other Beers Criteria drugs appeared to be 2% lower among ADRD group compared with inpatients without ADRD (aRR: 0.98 (95% CI: 0.97, 0.99), p<0.004). Clinicians need to be aware of potential side effects of using CNS Beers drugs in people with ADRD and whether there are alternatives to their use.

